# Late effects among colorectal cancer survivors ≥5 years after diagnosis—a systematic scoping review

**DOI:** 10.1093/jncics/pkag057

**Published:** 2026-06-12

**Authors:** Toktam Pour, Mary Jose Urruchua Rodriguez, Melissa S Y Thong, Hermann Brenner, Volker Arndt, Michael Hoffmeister

**Affiliations:** Division of Clinical Epidemiology of Early Cancer Detection, German Cancer Research Center (DKFZ), Heidelberg, Germany; Unit of Cancer Survivorship Outcomes & Epidemiology, German Cancer Research Center (DKFZ), Heidelberg, Germany; Medical Faculty, University of Heidelberg, Heidelberg, Germany; Division of Clinical Epidemiology of Early Cancer Detection, German Cancer Research Center (DKFZ), Heidelberg, Germany; Medical Faculty, University of Heidelberg, Heidelberg, Germany; Unit of Cancer Survivorship Outcomes & Epidemiology, German Cancer Research Center (DKFZ), Heidelberg, Germany; Cancer Prevention Graduate School, German Cancer Research Center (DKFZ), Heidelberg, Germany; Unit of Cancer Survivorship Outcomes & Epidemiology, German Cancer Research Center (DKFZ), Heidelberg, Germany; Division of Clinical Epidemiology of Early Cancer Detection, German Cancer Research Center (DKFZ), Heidelberg, Germany

## Abstract

**Background:**

Colorectal cancer (CRC) survival is improving, yet long-term survivors remain at risk for cancer- and treatment-related late effects, which may emerge many years after diagnosis and treatment. We aimed to summarize evidence on late effects and their risk factors reported ≥5 years after CRC diagnosis, compared to cancer-free controls.

**Methods:**

The literature search for this scoping review was conducted in PubMed, EMBASE, and Web of Science from inception to November 18, 2025. Primary research studies investigating late effects and providing relative risk estimates specifically ≥5 years after diagnosis were included. Risk of bias was assessed with the Newcastle-Ottawa-Scale. The study was registered with Open Science Framework, 10.17605/OSF.IO/JY6DX.

**Results:**

We identified 9 observational studies that reported relative risk estimates for late effects. Seven studies had a low risk of bias and 2 were at moderate risk. The most frequently investigated late effect was incidence of diabetes (5 studies). The highest relative risks were reported for immunity disorders (5-10 years hazard ratio [HR] = 4.56, 99% CI = 1.36 to 15.31), depression (>5 years HR = 2.65, 95% CI =1.61 to 4.36), and nutritional deficiency (5-10 years HR = 1.69, 99% CI = 1.38 to 2.08). Comorbidities at diagnosis, age at diagnosis, and treatment were the most common risk factors associated with late effects.

**Conclusions:**

This scoping review found that even ≥5 years after diagnosis late effects requiring treatment are considerably more common among CRC survivors than cancer-free controls. The small number of studies and the broad spectrum of identified late effects highlight the need for extensive research to characterize late effects to improve long-term survivorship care.

Box 1. Summary of key findings, gaps, and future research directions

**Key findings** 

- An increased risk of clinical depression and Type 2 diabetes was observed
- Evidence suggests a wide range of further late effects (immune, cardiovascular, and bone disorders) but findings are limited
- Current survivorship guidelines do not adequately address late effects

**Research gaps** 

- Scarcity of long-term CRC-specific studies examining outcomes emerging 5 years or later
- Heterogeneous outcome measures and definitions across studies, limiting comparability
- Inadequate control for key confounders, such as treatment exposure, disease stage, pre-existing conditions, and lifestyle behaviors

**Future research directions** 

- Establishing long-term CRC survivor cohorts to better distinguish late effects from earlier outcomes
- Harmonizing late effect definitions and assessment methods
- Developing risk stratification models to identify survivors at highest risk for late effects
- Investigaing mechanistic links between CRC and its treatments to late morbidity
- Generating evidence to support CRC-specific late-effect monitoring and survivorship care guidelines

## Introduction

**Figure 1.**
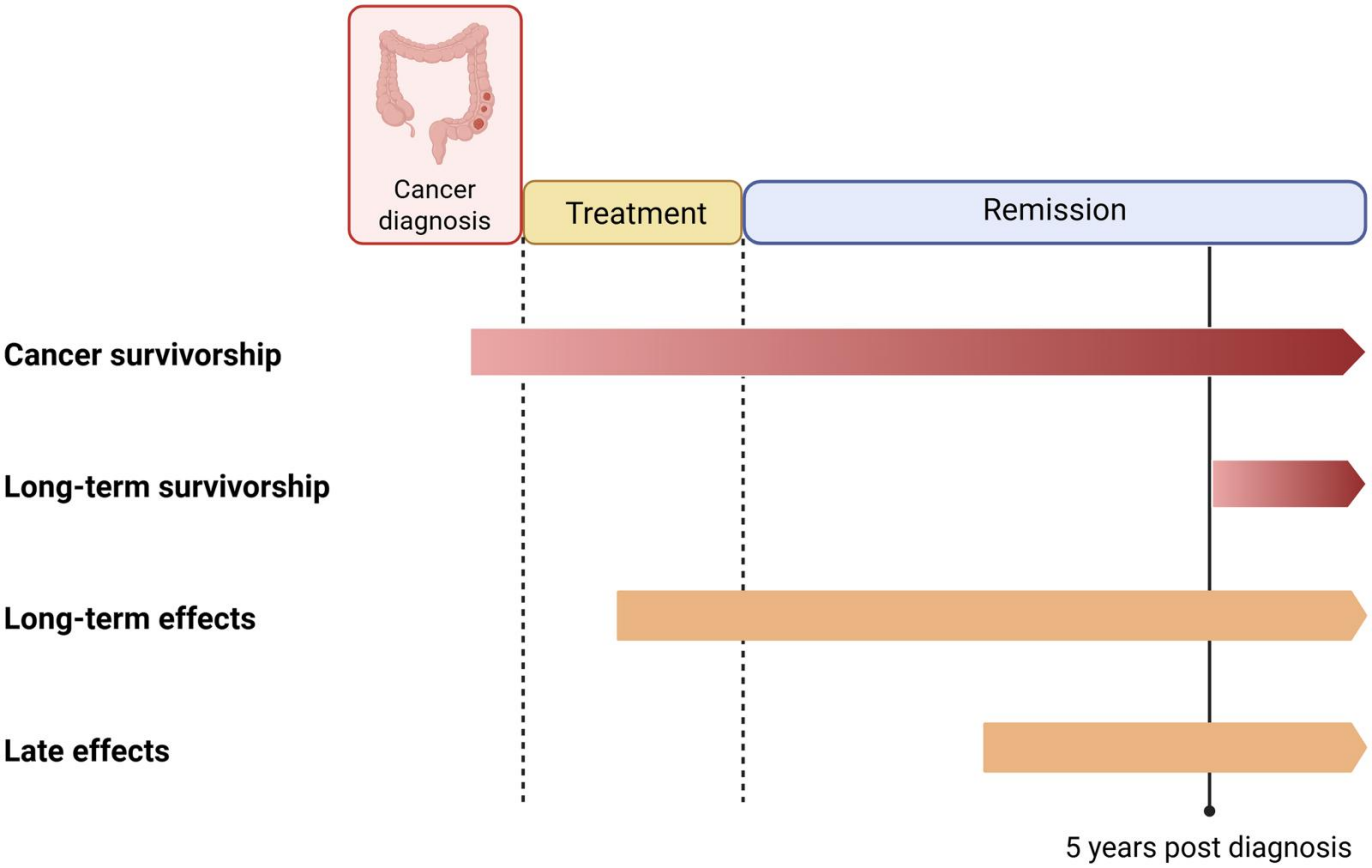
Timeline of survivorship terms. Created in BioRender. Hoffmeister, M. (2026) https://BioRender.com/3wxnpv2.

Colorectal cancer (CRC) is the third most commonly diagnosed cancer in the world and the number of CRC survivors is rising due to demographic ageing and improvements in diagnosis and treatment.^1^^,^^2^ According to a widely used definition, survivorship begins at the time of diagnosis and continues throughout a patient’s lifetime.^3^ Individuals living with a cancer diagnosis for 5 years or more are considered long-term survivors and represent around 65% of all CRC survivors in the US and 58%-83% in Europe^2^^,^^4^^,^^5^ (Figure 1). Although many of these long-term survivors transition from oncologist visits back to primary care, they continue to need health care that goes beyond those without cancer. Comprehensive survivorship care is still widely lacking in many countries.^6^^,^^7^

Challenges in long-term survivorship include, besides managing recurrence and new cancers, identifying and managing cancer- or treatment-related long-term and late effects.^8-10^ Long-term effects are adverse effects that manifest during or shortly after treatment and can persist for many years, like fatigue and chronic pain.^11^^,^^12^ On the other hand, late effects such as cardiovascular or metabolic diseases occur months or years after treatment has been completed^13^^,^^14^ (Figure 1). Whereas these definitions clearly differentiate between long-term and late effects, a unanimous differentiation may be difficult in individual survivors and even more across studies.

Despite the high relevance of identifying and diagnosing late effects, research and evidence remain still very limited with most studies focusing on short-term follow ups and long-term effects.^13^^,^^15^ This is partly because patients are more closely medically monitored in the first years after treatment, which facilitates identifying and managing long-term effects.^16^ Among the most prevalent long-term problems of CRC survivors are body image issues, peripheral neuropathy, gastrointestinal problems, and psychological distress, while insomnia and fatigue are often reported as the most severe.^17-19^ These persistent symptoms make it more difficult for survivors to adhere to lifestyle recommendations linked to better survival and quality of life.^20^ Lower rates of contacts between health care providers and survivors in later years of survival make it harder to document late effects and link them to the preceding history of cancer and cancer treatment, especially since primary care physicians often report uncertainty about cancer- and treatment-related health problems.^9^

Nevertheless, in recent years more studies including reports on late effects and with longer follow up have been conducted. These late effects are often age-associated comorbidities that occur earlier in cancer survivors compared to cancer-free individuals due to treatment-related accelerated biological aging.^21^^,^^22^ Furthermore, CRC is increasingly affecting younger adults (<50 years) in high-income Western countries.^23-25^ Therefore, with improving survival, understanding late effects in the context of CRC becomes crucial, especially for younger survivors, who face extended periods of survivorship.

**Figure 2.**
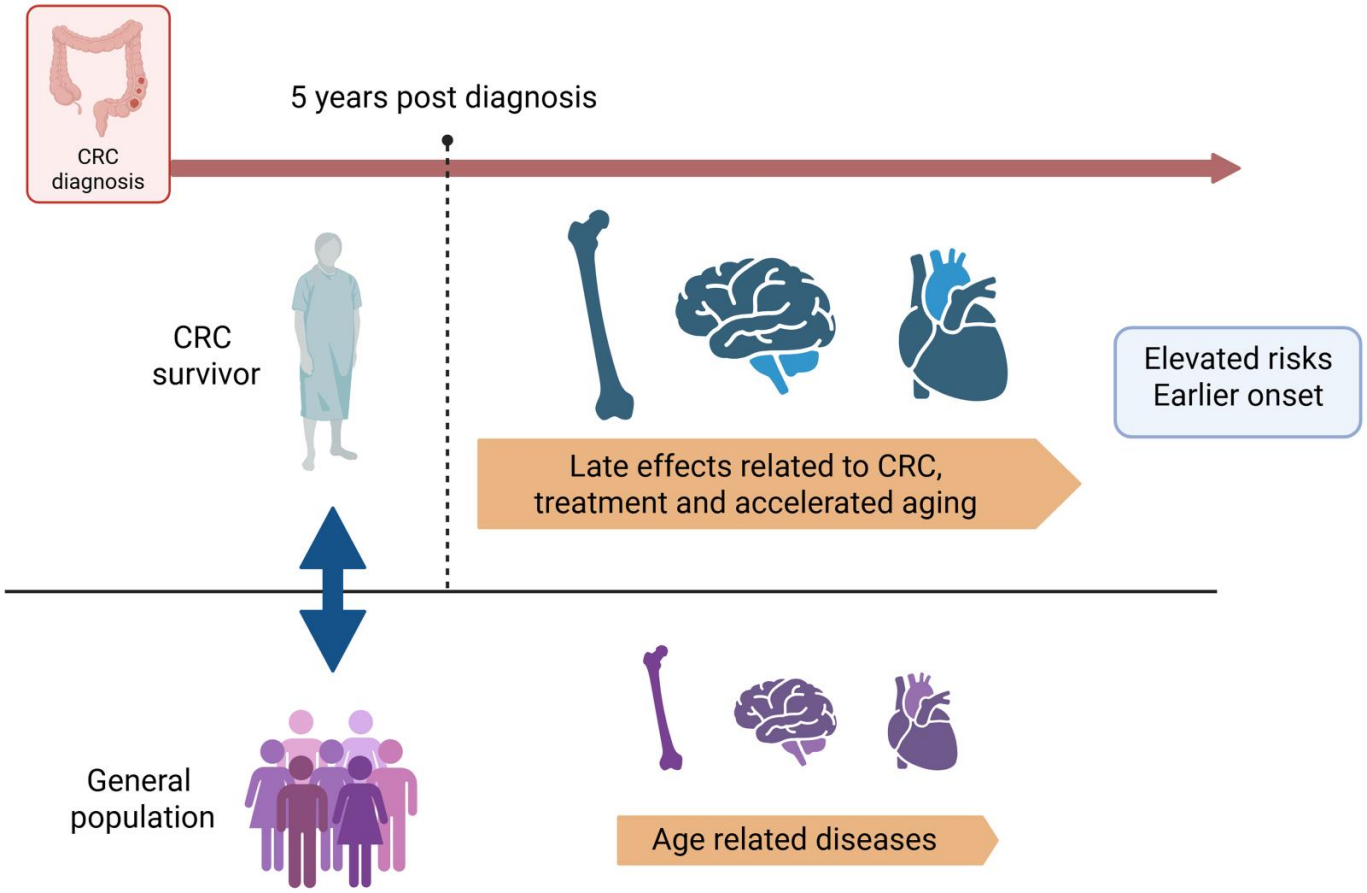
Study design and aim of this review. Created in BioRender. Hoffmeister, M. (2026) https://BioRender.com/5c9ok7f.

The aim of the review was to summarize existing evidence on late effects reported ≥5 years after CRC diagnosis and their risk factors, and to identify the knowledge gaps in this field (Figure 2).

## Methods

This systematic scoping review is reported according to the Preferred Reporting Items for Scoping reviews and Meta-Analyses (PRISMA) extension for scoping reviews (PRISMA-ScR) guidelines and is registered on Open Science Framework (OSF) with the registration DOI 10.17605/OSF.IO/JY6DX.

### Search strategy and study selection

A systematic search was conducted in PubMed, EMBASE, and Web of Science from inception until November 18, 2025 without restrictions on publication year. The detailed search strategy is provided in Table S1. The references of included studies, relevant reviews, and related publications were also utilized to identify additional studies.

The applied inclusion criteria were: (1) primary research studies (original articles) investigating late effects after CRC diagnosis with a follow up time of ≥5 years after diagnosis, (2) separate analysis for the time frame ≥5 years after diagnosis, (3) cancer-free control groups (4) if other cancer sites were included, a separate analysis for CRC, (5) age at diagnosis ≥18 years, and (6) published in a peer reviewed journal in English language.

The applied exclusion criteria were: (1) case studies and not primary research studies (reviews and commentaries), (2) studies collecting late effects only through patient-reported outcomes (PROs), (3) studies reporting cause of death outcomes but no other late outcomes.

After removing duplicate studies, the title and abstract of articles were screened by 1 reviewer (T.P.) according to the inclusion and exclusion criteria. In cases of uncertainty at this stage, articles were retained and progressed to full-text review to minimize the risk of excluding potentially relevant studies. Full-text screening was performed by 2 reviewers (M.J.U.R. and T.P.) independently. Any differences were resolved by discussion or by consulting further reviewers (V.A., M.H., and M.T.).

### Data extraction and study quality assessment

Data extraction and risk of bias assessment were conducted by 2 reviewers independently (M.J.U.R. and T.P.) using a predetermined extraction sheet. Information was collected on study characteristics (authors, publication year, country, title, study design, aim, sample size, follow-up, data source), study population (age at diagnosis, sex distribution, cancer site and stage, treatment), and data related to late effects (late effect, risk estimate, tool/method of assessment, impacting factors). We collected data from adjusted models, if available, and confidence intervals when applicable.

The Newcastle-Ottawa Scale (NOS) was used to evaluate the quality of participant selection, study comparability, and the assessment of outcome in all included studies (Table S2).^26^ The NOS guiding questions were adjusted for the use of this review and the criteria “Adequacy of follow up of cohorts” was changed to determine if the reported follow up time is CRC specific (Table S2). The NOS score ranges between 0 and 9: studies scoring 8-9 are considered high quality, those with 5-7 are classified as intermediate quality, and scores below 5 indicate low quality with a high risk of bias.

The meta package in R (version 4.5.0) was used to summarize and plot the outcomes.

## Results

### Study selection

**Figure 3.**
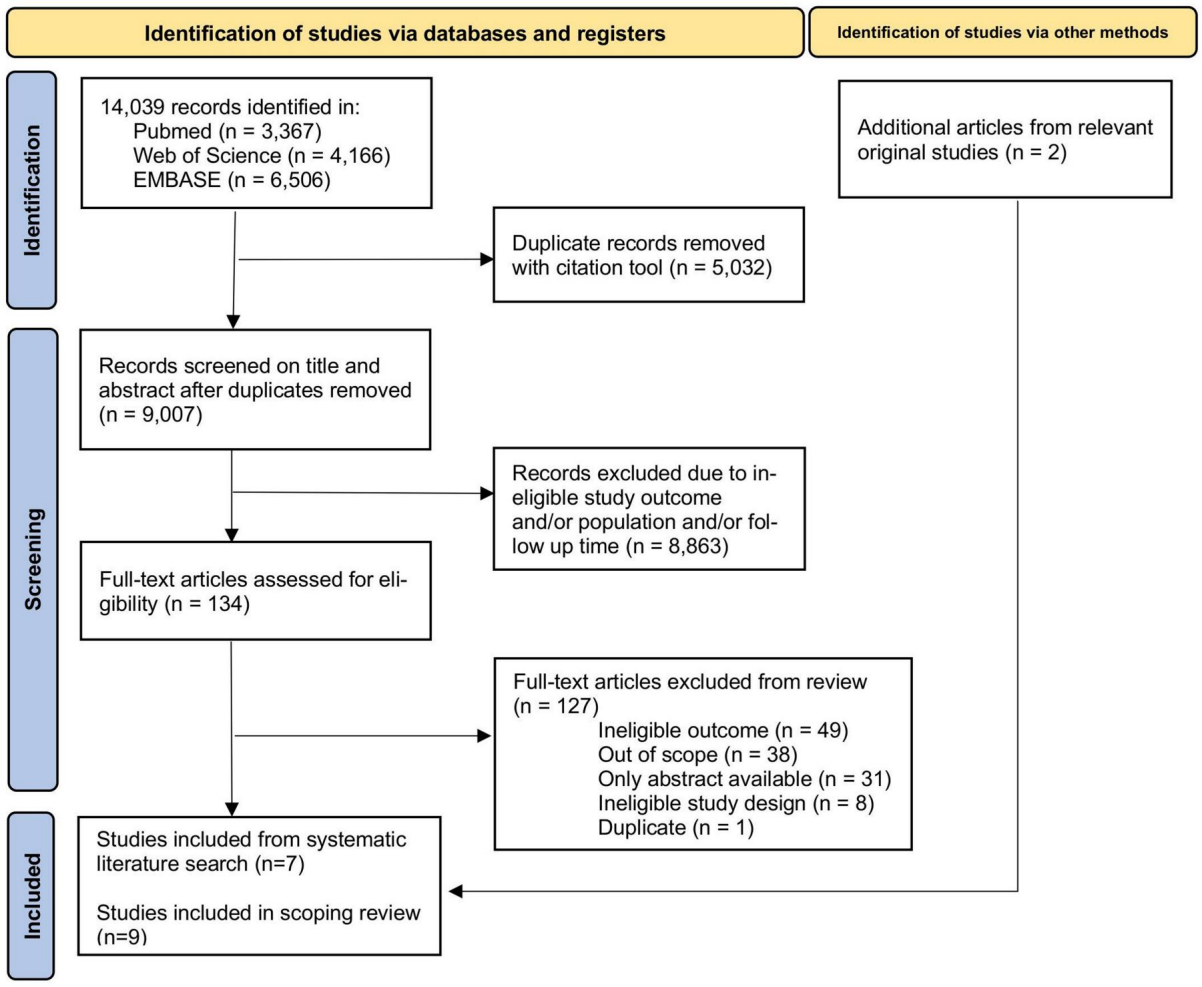
Flow diagram of literature search process.

After deduplication of the initial 14 039 identified records from the search 9007 records were screened on title and abstract (Figure 3). One hundred and forty-four articles were then full-text screened for eligibility, of which 7 articles met the inclusion criteria for the review. Most studies were excluded due to an ineligible outcome (*n* = 49, long-term effects or quality of life, comorbidities before diagnosis, cause of death), others were out of scope (*n* = 38, no specific results for CRC, follow up <5 years, no specific reporting of outcomes ≥5 years after diagnosis). Further studies were excluded because only the abstract was available (*n* = 31), used an ineligible study design (*n* = 8, case reports, reviews), or were duplicates (*n* = 1). Two additional articles were identified through cross-referencing and scanning related publications, which sums up to 9 final articles included in this review.

The main characteristics of the included studies are summarized in Table 1. Most studies were published within the last decade (*n* = 8). The majority of the studies were database or registry based (*n* = 6). Except for one study conducted in an upper-middle-income country (China = 1), all studies originated from high-income regions (United States = 3, United Kingdom = 2, Canada = 1, Denmark = 1, Germany = 1). The sample size ranged between 506 and 39 707, and the average age at diagnosis ranged between 62 and 68 years. The proportion of female survivors ranged between 31% and 73%. Three studies included survivors of all cancer stages, 1 study only cancer stages II-III, 1 only stages I-III, and 4 studies did not report cancer stage. All studies included colon and rectal cancer sites. The reported treatments included surgery, chemotherapy, radiotherapy, hormone therapy, and immuno-/antibody therapy. The NOS scores ranged between 6 and 9 and implied rather low risks of bias among the studies. Seven studies scored 8 points or higher and can be considered as high quality and the remaining 2 studies were of intermediate quality with a score of 6 points. Most studies lacked quality of outcome assessment (Table S3).

**Table 1.** Characteristics of included studies.

Author, year, country	Study design,	Sample size CRC (% female)	Cancer site (% of sample size)	Age (years)	Cancer stage	Reported treatments	Data source	Late effects	NOS Score
Yang et al. (2024), Germany^27^	Case-control	506 (31)	CRC	–	I-IV	Surgery, chemo-, radio-, immuno-/antibody therapy	CAESAR	Diabetes	6
Andresen et al. (2023), UK^28^	Database derived case-control	20 340 (45.5)	CRC	Median 73^b^	–	Surgery	CPRD/HES-APC	Acute kidney injury	9
Lee et al. (2022), China^29^	Database derived case-control	1037 (36)	CRC	Median 62^a^	II-III	Adjuvant chemotherapy	CDARS	CVDs	9
Zeng et al. (2022), USA^30^	Cohort study	3402 (73)	Colon (43%) and rectal (57%) cancer	Mean 66.7^c^	I-III	–	NHS, NHS II, HPFS	Type 2 diabetes	6
Kjaer et al. (2021), Denmark^31^	Case-control	1324 (43)	Colon (66%) and rectal (34%) cancer	Mean 71.3^b^	I-IV	Surgery, chemo-, radiotherapy	DCH	Depression	8
Hawkins et al. (2019), USA^32^	Database derived case-control	7114 (48)	CRC	Mean 63.7^a^	–	–	UPDB	Endocrine and metabolic diseases	8
Lloyd et al. (2019), USA^33^	Database derived case-control	8961 (48)	Colon (75%) and rectal (25%) cancer	–	I-IV	Surgery, chemo-, radiotherapy	UPDB	Mental health disorders	9
Singh et al. (2016), Canada^34^	Registry based case-control	39 707 (47)	Colon (69%) and rectal cancer (31%)	Mean 68^a^	–	Chemotherapy	OCR	Diabetes	8
Khan et al. (2011), UK^35^	Database derived case-control	5068 (49)	CRC	Mean 74.1^b^	–	Surgery, chemo, and/or radiotherapy, and hormone therapy	UK GPRD	Diabetes, dementia, osteoporosis	9

a Age at diagnosis.

b Age at study entry.

c Age all 3 cohorts combined.

Abbreviations: CAESAR = Cancer Survivorship—a multi-regional population-based study; CDARS = Clinical Data Analysis and Reporting System; CPRD/HES-APC = Clinical Practice Research Datalink/Hospital Episode Statistics Admitted Patient Care; DCH = Diet, Cancer and Health study; GPRD = General Practice Research Database; HPFS = Health Professionals Follow-Up Study; NHS = Nurse Health Study; OCR = Ontario Cancer Registry; UPDB = Utah Population Database.

### Late effects

**Figure 4.**
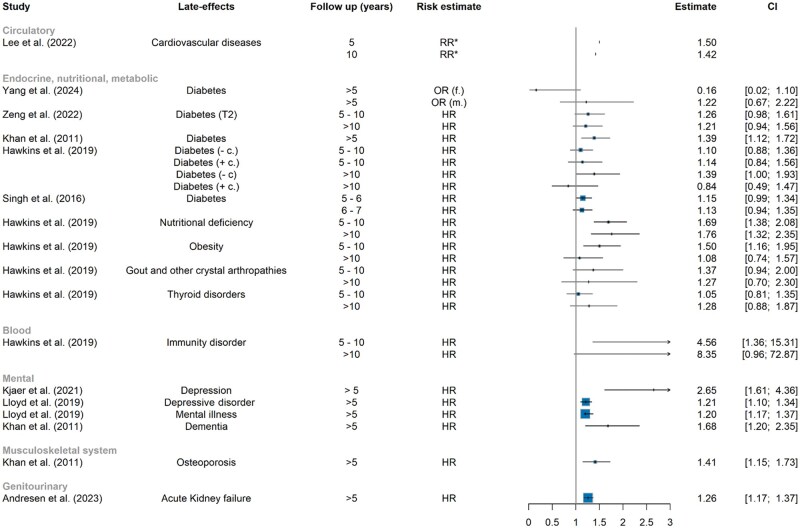
Studies reporting relative risks for late effects occurring ≥5 years after CRC diagnosis sorted by diagnostic group. * = risk ratios approximated based on cause-specific cumulative incidences provided in the publication. Abbreviations: − c. = without complication; + c. = with complications; T2 = Type 2.

The studies reported a wide range of late effects by using heterogeneous statistical methods. To summarize the late effects, we constructed a forest plot showing the studies reporting relative risks (Figure 4). Two studies reported relative risks for a large number of diagnostic groups.^32^^,^^33^ From those studies, only late effects with high and significant risk increase or a clear diagnostic term were chosen for visualization. The follow-up time ranged from 5 years up to more than 10 years.

Of the wide variety of late effects that were investigated in the studies, the late effects reported by more than 1 study were diabetes (5 studies: ^27^^,^^30^^,^^32^^,^^34^^,^^35^) and depression or depressive disorder (2 studies: ^31^^,^^33^).

Compared to controls without a cancer history, CRC survivors had much higher risks for immunity disorders (5-10 years HR = 4.56, 99% CI = 1.36 to 15.31; >10 years HR = 8.35, 99% CI = 0.96 to 72.87), depression (>5 years HR = 2.65, 95% CI =1.61 to 4.36), and nutritional deficiency (5-10 years HR = 1.69, 99% CI =1.38 to 2.08; >10 years HR = 1.76, 99% CI =1.32 to 2.35).^31^^,^^32^

The relative risks for late effects investigated at different time points within a study are higher in earlier years after diagnosis, compared to later years except for nutritional deficiencies, thyroid disorder, and immunity disorders.

### Risk factors

The most common risk factors found to be associated with the reported late effects were previous comorbidities, age, and treatment. Comorbidities and an older age at diagnosis seemed to play a role in cardiovascular diseases (CVD), depression, and other mental illnesses.^29^^,^^31^^,^^33^ Survivors of younger age seemed to have a higher relative risk for endocrine, nutritional, and metabolic diseases, and for acute kidney failure.^28^^,^^30^^,^^32^ Chemotherapy was found to be associated with a higher risk of CVD and a lower relative risk of diabetes.^29^^,^^34^ Radiotherapy was associated with a higher risk of depression.^31^

## Discussion

To the best of our knowledge, this is the first systematic scoping review that comprehensively addresses late effects at least 5 years after diagnosis in long-term CRC survivors with the clear aim to distinguish from adverse effects occurring in earlier cancer survivorship. The key findings and gaps identified in this review are summarized in Box 1. Overall, a broad spectrum of late effects occurring ≥5 years after diagnosis across multiple diagnostic groups were identified by different studies. Of all late effects, only depression and diabetes were reported by more than one study and with heterogeneous outcome measures.

Depression and anxiety are frequently reported years after cancer diagnosis.^36^ We found 2 studies providing relative risk estimates for clinical depression among CRC long-term survivors, both reporting elevated risks, which implies the need for medical attention and treatment of this late effect as part of CRC survivorship care.^31^^,^^33^ This outcome corresponds with the results of another study which also investigated clinical depression in survivors of different cancer entities and found an elevated risk for depression in long-term survivors.^37^ In contrast to clinical depression, which usually needs to be diagnosed by a professional, depressive symptoms can be assessed with PROs and are well studied in cancer survivors.^38^ A recent systematic review on symptoms of depression in long-term cancer survivors found no difference in prevalence rates in comparison to cancer free individuals and concluded that there is no need to actively screen for depression in long-term cancer survivors.^39^ However, this blanket conclusion may not be applicable to long-term CRC survivors as our review identified an increased risk of clinical depression in survivors with comorbidities compared to non-cancer controls. Further research is necessary to determine if adapted screening for clinical depression should be applied in the context of CRC. Furthermore, another study investigating age and sex specific subgroups of long-term survivors and their risk for depressive symptoms 5 and 10 years after survival found that survivors of younger age and females have higher risks.^40^ This stresses the importance of and need for research to determine possible risk factors for depression which could be assessed by clinicians to identify patients that may be specifically in need of screening. Psychosocial challenges that have been detected in cancer survivors and which seem to enhance depression risk include fear of recurrence, unemployment, financial difficulties, body image disturbance, and coping with physical long-term effects like fatigue and sexual dysfunction.^41^^,^^42^ Whether these challenges are also risk factors for clinical depression in long-term CRC survivors needs to be investigated alongside other possible factors.

A number of studies included in this review found that the risk of developing diabetes was higher among long-term CRC survivors than in the general population.^27^^,^^30^^,^^32^^,^^34^^,^^35^ Considering the advanced age of the cohort, it is reasonable to assume that almost all diabetes cases correspond to type 2 diabetes (T2D). Although most of the reported relative risks were not statistically significant, a meta-analysis^30^ with studies published up to May 2021 reported a statistically significant increased risk for T2D within the first 10 years after CRC diagnosis (≤5 years HR = 1.32, 95% CI = 1.27 to 1.36; 5.1 to 10 years HR = 1.14, 95% CI = 1.04 to 1.25) and non-significantly increased risk for >10 years (HR = 1.14, 95% CI = 0.91 to 1.37). These findings indicate that the increased relative risk for T2D declines over the years. Therefore, it may be relevant to incorporate T2D screening for certain subgroups of long-term CRC survivors. Although some of these cohort studies included large sample sizes, the number of T2D cases often remained limited which hindered subgroup analyses.^30^ Furthermore, some of these studies either did not adjust for well-known risk factors of T2D such as body mass index, lifestyle factors (eg, diet and smoking), family history, and other metabolic and clinical factors or only took body mass index into account^32^^,^^34^^,^^35^, limiting the interpretability of results.

Moreover, the mechanisms by which CRC or its treatments might affect the development of diabetes are still unclear. Possible pathways include inflammation, gut microbiota alterations, and impairment of glucose metabolism through chemotherapy.^43-46^ Considering the complex, bilateral relationship between metabolic dysfunction and cancer, additional studies are needed to clarify how diabetes develops in CRC patients. Additionally, a recent study investigating the risk of T2D among older Asian, native Hawaiian, and Pacific Islander CRC survivors found disproportionately higher risks of T2D in comparison to non-Hispanic White CRC survivors and especially for Southeast and East Asian survivors.^47^ This stresses the importance of considering ethnic differences in future research in this field.

Furthermore, elevated risks for a range of other late effects have been found.^28^^,^^29^^,^^32^^,^^33^^,^^35^ The late effect with the highest relative risk in this review is the International Classification of Diseases (ICD) -9 term “immunity disorder,” which includes immune deficiency disorders and autoimmune disorders.^32^ Recent studies showed that cancer burden and conventional treatments such as surgery, chemotherapy, and radiation may profoundly disrupt systemic immune organization and reshape circulating immune cells.^48^ These alterations may be the cause of the detected immunity disorders in long-term survivors and may contribute to further late effects. Therefore, future survivorship research should aim to explore this area in greater depth and detail with a range of possible immune disorders.

Late effects that have been investigated in the context of CRC in studies with follow ups of ≥5 years, which did not report specific risk estimates for long-term survivors compared to a control population, included pelvic fractures in rectal cancer survivors who underwent radiotherapy.^49^^,^^50^ Although radiotherapy improved clinical outcomes, it has been found to adversely affect bone metabolism by impairing osteoblast function, reducing bone vascularization, and decreasing bone density.^51^ Such late effects after radiotherapy could also be related to the occurrence of osteoporosis among CRC long-term survivors as reported by Khan et al.^35^ Further research investigating bone density and fracture risk in long-term CRC survivors undergoing different treatments is needed in order to understand these associations better.

Late effects that have been increasingly investigated for survivors of various cancer entities are CVD.^52^ However, the only study reporting CVD risk estimates as specifically for ≥5 years after CRC diagnosis is the study by Lee et al., which presented cause-specific cumulative incidences but also showed overall relative CVD risk since CRC diagnosis (HR = 2.11, 95% CI = 1.39 to 3.20).^29^ When considering further studies with a follow up of ≥5 years after diagnosis which reported relative risks for the entire follow-up period only, we noted more indications for elevated CVD risks in CRC survivors: Kenzik et al. observed increased risks for CVD (HR = 3.07, 95% CI = 2.98 to 3.16) and congestive heart failure (HR = 4.19, 95% CI = 4.06 to 4.33) for survivors followed up to 10 years after diagnosis^53^ Another study by Florido et al. with 5.1 years of follow-up in the CRC subcohort also showed elevated CVD risks in comparison to cancer-free controls (HR = 1.46, 95% CI = 1.15 to 1.85).^54^ However, this association was not statistically significant after excluding participants who died within 1 year of cancer diagnosis. On the other hand, one study could not find higher relative risks for CVDs in survivors of colon or rectal cancer when investigating multiple cancer entities in a cohort study.^55^ Furthermore, there are also some studies presenting higher incidence rates for CRC survivor subgroups undergoing radiotherapy or making unhealthy lifestyle choices like smoking and drinking alcohol.^56^^,^^57^ A recent study investigated CVDs in long-term survivors of multiple cancer entities, including CRC.^58^ Interestingly, that study reported strong associations with non-cancer related factors such as older age, male sex, and comorbidities, but not for cancer-related factors such as cancer therapy. These findings are contradictory to the known cardiotoxic traits of treatments like chemotherapy and radiation.^59^^,^^60^ The study by Lee et al. included in this review found associations with chemotherapy and CVD risk alongside comorbidities and older age, but these analyses were not restricted to the time ≥5 years after diagnosis.^29^ Another recent study investigated CVD risk in CRC survivors with and without metabolic syndrome and found that metabolic syndrome was associated with elevated CVD risk.^61^ This stresses the importance of comorbidities in the context of late effects. Overall, future research is needed to determine whether the relative risk for CVD is truly elevated in long-term survivors and to provide deeper insights into risk factors such as chemotherapy and comorbidities.

The study by Chang et al. comprehensively evaluated the burden of 144 conditions across multiple cancer entities, including CRC, by employing electronic health record datasets.^62^ The top 5 health conditions with the biggest increase in cumulative burden between 5 and 10 years post diagnosis were hypertension, diabetes, chronic obstructive pulmonary disease, coronary heart disease (not otherwise specified), and atrial fibrillation. The highest relative risk estimates reported irrespective of time since diagnosis were septicemia, agranulocytosis, acute kidney injury, pleural effusion, and meningitis. These outcomes point to additional possible late effects of CRC that should be investigated in the future. In another study, the severity of late effects in long-term CRC survivors has been investigated utilizing patterns of hospitalizations due to somatic diseases in cancer survivors compared with cancer-free individuals, and found elevated relative risk for CRC survivors for hospitalization across all 11 main diagnostic groups of the ICD-10 system.^63^

The majority of the included articles in this review were registry-based. Therefore, most late effects were determined in big sample sizes with medical records utilizing ICD codes as standardized outcome measures, ensuring good data quality which corresponds with the high NOS scores.

On the other hand, most included studies also investigated earlier years of survivorship and did not specifically address survivorship 5 years or longer. This emphasizes the need for more research specifically in cohorts of long-term CRC survivors. Furthermore, although some possible risk factors were found to be associated with the incidence of late effects in this review, more data on possible important confounders and risk factors like cancer stage and CRC subsite are missing in multiple studies. In order to provide tailored survivorship care in the future, as recommended by experts, it would be necessary to identify possible risk differences between distinct survivor groups due to for example, cancer treatment, and to assess the interactions with quality of life and lifestyle.^64^ Therefore, future studies should collect more comprehensive information on cancer related data. Further gaps in research include the identification of survivors with the highest risk of developing late effects and investigations into effective preventive measures.

Although late effects are a growing concern for long-term CRC survivors and the healthcare sector as a whole, the American Cancer Society Colorectal Cancer Survivorship Care guidelines mainly focus on cancer recurrence and persistent long-term effects.^65^ These guidelines also highlight the limited evidence and the need for survivorship care plans to ensure quality of long-term care. The European Society for Medical Oncology also only addresses recurrence and persistent long-term effects such as bowel dysfunction and psychological distress rather than surveillance for late effects and recommendations for healthy lifestyle promotion.^66^^,^^67^ More recently, the National Comprehensive Cancer Network patient guidelines for cancer-related late and long-term effects expanded this view by providing overarching guidance specifically for late effects like heart disease, but these new guidelines are still limited and not cancer site specific.^68^ The evidence of late effects in long-term survivors of CRC summarized in this systematic scoping review highlights the need for more extensive CRC specific recommendations to equip primary care clinicians for managing late effects and to ensure sufficient long-term survivorship care in the future.

This review has strengths and limitations that should be considered. One of the strengths is the systematic search for relevant publications, and the differentiation of late effects ≥5 years after diagnosis, which have not been summarized before and which reduces the potential overlap of long-term and late effects.

Limitations of our work comprise possibly underestimating the late effect burden in CRC survivors by missing late effects which occur 1-4 years past diagnosis and by not incorporating studies that did not provide separate risk estimates for 5 years and later. Furthermore, studies investigating late effects ≥5 years after CRC diagnosis are scarce. Although this is a limitation, it also clearly shows the need for additional studies in long-term survivors.

## Conclusions

Overall, in this systematic scoping review we found that CRC survivors have a greater risk for developing chronic diseases ≥5 years after diagnosis in comparison to the general population, including diabetes, clinical depression, immune disorders, and renal failure. Nevertheless, more research is required with longer follow-ups of CRC patients to strengthen the evidence on the occurrence of late effects, to identify high risk patients and evaluate effective preventive measures, and therefore to enable improved survivorship care for long-term CRC survivors.

## Supplementary Material

pkag057_Supplementary_Data

## Data Availability

Since this is a review, no new data were generated or analyzed for the manuscript.
