# Supplementary material for: Late effects among colorectal cancer survivors ≥5 years after diagnosis—a systematic scoping review
Source: JNCI Cancer Spectr. 2026 Jun 12;10(3):pkag057. doi: 10.1093/jncics/pkag057 (PMC13310035; doi:10.1093/jncics/pkag057)
Supplement: pkag057_Supplementary_Data [file pkag057_supplementary_data.docx]

**Supplementary Materials:**

**Late effects among colorectal cancer survivors ≥5 years after diagnosis– A systematic scoping review**

Toktam Pour M.Sc^1,2,3^, Mary Jose Urruchua Rodriguez M.Sc^1,3^, Melissa S. Y. Thong PhD^2^, Hermann Brenner Prof. MD^4^, Volker Arndt Prof. MD^2*^, Michael Hoffmeister Prof. MD^1*^

1. Division of Clinical Epidemiology of Early Cancer Detection, German Cancer Research Center (dkfz), Im Neuenheimer Feld 280, 69120 Heidelberg, Germany

2. Unit of Cancer Survivorship Outcomes & Epidemiology, German Cancer Research Center (dkfz), Im Neuenheimer Feld 280, 69120 Heidelberg, Germany

3. Medical Faculty, University of Heidelberg, Im Neuenheimer Feld 672, 69120 Heidelberg, Germany

4. Cancer Prevention Graduate School, German Cancer Research Center (dkfz), Im Neuenheimer Feld 280, 69120 Heidelberg, Germany

* Shared last authorship

**Table of Contents**

Table S1: Search strategy

Table S2: Newcastle - Ottawa Quality Assessment Scale

Table S3: Newcastle - Ottawa Quality Assessment Scale scores of included studies

**Table S1: Search strategy**

|  | **Pubmed** | **EMBASE** | **Web of Science** |
| --- | --- | --- | --- |
| #1 | (("colo* cancer"[tiab] OR "rectal cancer"[tiab] OR "rectal carcinoma"[tiab] OR "colo* carcinoma"[tiab]) OR CRC[tiab] OR "colorectal neoplasm*"[tiab] OR "Colorectal Neoplasms"[mesh])  *Number of records: 335*,775 | ('colo* cancer':ab,ti OR 'rectal cancer':ab,ti OR 'rectal carcinoma':ab,ti OR 'colo* carcinoma':ab,ti OR crc:ab,ti OR 'colorectal neoplasm*':ab,ti OR 'colorectal tumor':ab,ti OR 'colorectal tumor'/exp/mj)  *Number of records: 436,307* | "colo* cancer" OR "rectal cancer" OR "rectal carcinoma" OR "colo* carcinoma" OR CRC OR "colorectal neoplasm*" (Topic)  *Number of records: 473,359* |
| #2 | #1 AND (sequelae*[tiab] OR "health condition*"[tiab] OR "health effect*"[tiab] OR "health outcome*"[tiab] OR "adverse effect*"[tiab] OR "late effect*"[tiab] OR "late toxicit*"[tiab] OR comorbid*[tiab] OR "late complication*"[tiab] OR "long term adverse effect*"[tiab] OR "Long Term Adverse Effects"[mesh] OR Comorbidity[Mesh])  *Number of records: 8,910* | #1 AND (sequelae*:ab,ti OR 'health condition*':ab,ti OR 'health effect*':ab,ti OR 'health outcome*':ab,ti OR 'adverse effect*':ab,ti OR 'late effect*':ab,ti OR 'late toxicit*':ab,ti OR comorbid*:ab,ti OR 'late complication*':ab,ti OR 'long term adverse effect':ab,ti OR 'comorbidity'/exp/mj)  *Number of records: 13,524* | #1 AND sequelae* OR “health condition*” OR "health effect*" OR "health outcome*" OR “adverse effect*” OR “late effect*” OR “late toxicit*” OR comorbid* OR "long term adverse effect*" OR “late complication*” (Topic)  *Number of records: 8,162* |
| #3 | #2 AND ("Post diagnosis"[tiab] OR "Post treatment"[tiab] OR "Post cancer"[tiab] OR late[tiab] OR "Cancer survivor*"[tiab] OR Survivor*[tiab] OR Survival[tiab] OR "Cancer Survivors"[Mesh] OR longterm[tiab] OR "long-term"[tiab] OR "long term"[tiab] OR "follow up"[tiab] OR "follow-up"[tiab])  *Number of records: 4,274* | #2 AND ('post diagnosis':ab,ti OR 'post treatment':ab,ti OR 'post cancer':ab,ti OR late:ab,ti OR 'cancer survivor*':ab,ti OR survivor*:ab,ti OR survival:ab,ti OR longterm:ab,ti OR 'long-term':ab,ti OR 'long term':ab,ti OR 'follow up':ab,ti OR 'follow-up':ab,ti OR 'cancer survivor'/exp/mj)  *Number of records: 6,947* | #2 AND "Post diagnosis" OR "Post treatment" OR "Post cancer" OR late OR “Cancer survivor*” OR Survivor* OR Survival OR longterm OR “long-term” OR “long term” OR “follow up” OR “follow-up*" (Topic)  *Number of records: 4,410* |
| #4 | #3 NOT (child*[tiab] OR child[mesh])  Number of records: 4,*172* | #3 NOT ('child*':ab,ti OR 'child'/exp)  *Number of records: 6,818* | #3 NOT child* (Topic)  *Number of records: 4,335* |
| #5 | #4 Filters: English, Humans  *Number of records: 3,367* | #4 AND 'human'/de (Filter – study type – human)  *Number of records: 6,506* | #4 NOT animal* OR “animal experiment*” OR “animal model*” OR “animal tissue*” OR “non human” OR nonhuman OR rat OR rats OR mice OR mouse OR swine OR porcine OR murine OR sheep OR lambs OR pig OR pigs OR piglet* OR rabbit* OR monkey OR bovine (Topic)  *Number of records: 4,166* |

**Table S2:**

**NEWCASTLE - OTTAWA QUALITY ASSESSMENT SCALE**

**(Adapted for the outcomes of interest of this review)**

**Selection**

1) Representativeness and adequate definition of the exposed cohort/ cases (CRC)

a) truly or somewhat representative of the average CRC survivor **(1 point)**

b) selected group of users eg nurses, volunteers/ potential for selection biases (0 points)

c) no description or not stated (0 points)

2) Selection of the non exposed cohort/ controls

a) drawn from the same community as the exposed cohort/ community controls **(1 point)**

b) drawn from a different source/ hospital controls (0 points)

c) no description (0 points)

3) Ascertainment of cases (CRC) and/ or exposure (CRC treatment)

a) secure record (eg. medical records) **(1 point)**

c) based on self-reports (0 points)

d) no description (0 points)

4) Demonstration that outcome of interest (late effect/ comorbidities) was not present at start of study

a) yes **(1 point)**

b) no (0 points)

**Comparability**

1) Comparability on the basis of the design or analysis

if study includes CANCER FREE CONTROLS:

- study controls for sex, age, comorbidities at/ before diagnosis and at least 2 additional lifestyle factors (e.g. BMI, smoking, alcohol, socioeconomic status, medication) **(2 points)**
- study controls for sex, age, comorbidities at/ before diagnosis and at least 1 additional lifestyle factor (e.g. BMI, smoking, alcohol, socioeconomic status, medication) **(1 point)**
- study controls for sex or age and/or other factors (0 points)

**Outcome**

1) Assessment of outcome (late effect/ comorbidities)

a) independent assessment or record linkage **(1 point)**

c) self report (0 points)

d) no description (0 points)

2) Was follow-up long enough for outcomes to occur

a) yes >5 years for all participants or most participants (mean follow up time) **(1 point)**

c) yes, up to >5 years of follow up but not for most participants (mean follow up time<5 years) or

mean or median follow up time not reported (0 points)

3) Adequacy of follow up of cohorts

a) the follow up time was specifically assessed for CRC survivors (1 point)

b) the follow up time was only assessed for survivors of different cancer sides including CRC

survivors (0 points)

**Table S3: Newcastle - Ottawa Quality Assessment Scale scores of included studies**

| Author, Year | Selection | Comparability | Outcome | Total Score |
| --- | --- | --- | --- | --- |
| Yang et al. (2024), | 3 | 2 | 1 | **6** |
| Rutegard et al. (2023) | 4 | 2 | 2 | **8** |
| Andresen et al. (2023) | 4 | 2 | 3 | **9** |
| Chang et al. (2023) | 3 | 0 | 2 | **5** |
| Lee et al. (2022) | 4 | 2 | 3 | **9** |
| Zeng et al. (2022), | 2 | 2 | 2 | **6** |
| Kjaer et al. (2021) | 4 | 2 | 2 | **8** |
| Hawkins et al. (2019) | 4 | 1 | 3 | **8** |
| Lloyd et al. (2019) | 4 | 2 | 3 | **9** |
| Singh et al. (2016) | 4 | 2 | 2 | **8** |
| Khan et al. (2011), | 4 | 2 | 3 | **9** |
|  |  |  |  |  |
